# How to Improve Cancer Prevention Knowledge? A Way to Identify Gaps and Tackle the Limited Availability of Health Education Services in Primary Health Care Using the European Code Against Cancer

**DOI:** 10.3389/fpubh.2022.878703

**Published:** 2022-05-02

**Authors:** Monika Karasiewicz, Ewelina Chawłowska, Agnieszka Lipiak, Barbara Wiȩckowska

**Affiliations:** ^1^Laboratory of International Health, Department of Preventive Medicine, Poznan University of Medical Sciences, Poznań, Poland; ^2^Department of Computer Science and Statistics, Poznan University of Medical Sciences, Poznań, Poland

**Keywords:** health education, risk factors, health literacy, European Code Against Cancer, cancer prevention, family health history, primary health care, community-based participatory research

## Abstract

**Introduction:**

The first line of action against cancer is primary and secondary prevention. Increased efforts are needed in countries where cancer mortality is high and the healthcare system is inefficient. Objectives: Our aim was to present a new solution to identify and fill gaps in health education services in accordance with the European Code Against Cancer (ECAC).

**Materials and Methods:**

This study was carried out in a rural population of 122 beneficiaries of health education workshops financed by the Polish Cancer League. A self-developed questionnaire was used. PQStat v1.6.8. was also applied.

**Results:**

Our respondents were mostly farmers (53.3%) and manual workers (16.4%). Most participants self-assessed their health knowledge as good (46.7%). While 42% of all respondents claimed to know the healthy eating pyramid, only 8.2% correctly recognised all of its principles and 23.8% realised the importance of limiting the consumption of red meat. The most commonly recognised cancer risk factor were genetics (72.1%), stimulants such as alcohol or tobacco (51.5%) and environmental pollution (45.1%). UV radiation was not commonly recognised as a risk factor by respondents despite high occupational exposure in this population. We found a high percentage of male smokers. As many as 64.8% of respondents had not been counselled on cancer prevention in their clinics. A family history of cancer (FHC) did not differentiate respondents' health knowledge, health behaviors, or frequency of receiving cancer prevention counselling. Health education and health promotion in the region were unsatisfactory.

**Conclusions:**

Primary health care (PHC) should become more involved in promoting cancer prevention knowledge. One way could be to encourage health professionals to promote the ECAC. Cancer prevention should target especially persons with FHC and focus on modifiable cancer risk factors. At the workshops we were able to adjust the strength of each ECAC recommendation to best fit the target audience. By diagnosing and targeting specific communities, we can draw the attention of PHC staff and decision-makers to local health promotion needs, which is a good starting point for improving the situation. However, larger scale projects are needed to help design specific solutions to support primary healthcare in promoting ECAC.

## Introduction

Cancer incidence is reported to be on the increase globally (1–6). Also in Poland, the risk of developing cancer before the age of 75 is 30% for men and 24.5% for women, while the risk of dying from cancer is 18.7 and 11.7%, respectively (4). The increasing incidence has numerous determinants (1, 3, 5), including lifestyle-related modifiable ones (1, 2, 7, 8). Since treating cancer is challenging, we should make primary and secondary prevention the first line of action against cancer in Europe (6, 8). It is optimistically estimated that up to a half of all cancer cases could be prevented or avoided (2, 6, 7, 9–12). However, few Europeans live a healthy lifestyle (13).

Considering the overwhelming mass of available information on the prevention of lifestyle-related diseases, it is essential to inform communities about evidence-based preventive measures. Although knowledge alone will not suffice, it is necessary since one must first have knowledge before one can take action (14, 15). A set of 12 key recommendations reflecting current evidence on cancer prevention is presented in an accessible way in the 4th edition of the European Code Against Cancer (ECAC) (2, 6, 16). It is a European Commission initiative coordinated and endorsed by the International Agency for Research on Cancer. The ECAC is available in many official EU languages. For more than three decades, it has served as a tool for improving health literacy in the area of cancer prevention and health policy development (6, 12). Although health professionals report using the tool, the awareness of the ECAC in Europe is still low (6). Increased cancer prevention efforts are needed in countries from Central and Eastern Europe, including Poland, where cancer mortality is one of the highest in the region (2, 3) and the healthcare system is inefficient (17, 18). While Polish health-promoting organisations report using various strategies to disseminate ECAC recommendations (6), the effectiveness of these efforts must be evaluated in order to make any improvements.

Place of residence (urban vs. rural) is known to be associated with different exposures to certain risk factors (19–25), differences in access to health services (26, 27), and differing survival rates of cancer patients (28–30). Therefore, we decided to assess health knowledge and health behaviours referred to in the ECAC as well as the availability of health education in a population inhabiting a remote rural area. As employees of a large medical university which educates health professionals in Wielkopolska – the third most populous province in Poland – we needed feedback on the effectiveness of health promotion efforts from vulnerable populations inhabiting areas with poorer socioeconomic conditions and worse epidemiological indicators (31) in order to respond appropriately. It is particularly important given the fact that medical professionals in Poland seem inadequately prepared to provide preventive counselling to their patients (32, 33).

It is suggested that education targeted at rural populations should involve tailor-made activities (34) and that small-scale focused events are the most effective in terms of the number of individuals directly receiving education (35). We attempted to incorporate these principles while developing the project we are presenting in this study. We called the project “A Mobile Center for Health Education and Health Promotion in the Countryside”. The approach we used as its basis was community-based participatory research (CBPR), which consists in equitable collaboration of different stakeholders, in this case: the community, healthcare professionals, authorities, researchers, and a non-governmental organization. We chose the ECAC as a tool used in our health education and promotion efforts undertaken as part of the project.

## Objectives

The main aim of our study was to determine cancer education and prevention needs in a rural community and, consequently, to identify both the ECAC recommendations and the groups that ECAC promoters should pay special attention to.

We attempted to achieve the main objective through the accomplishment of the following specific objectives:

(i) analysing participant' self-assessed health knowledge, in particular their knowledge on cancer risk factors;
(ii) examining participants' reported health behaviours such as general taking care of their health, their physical activity, smoking, and alcohol use;
(iii) exploring participants' assessment of health education and health promotion activities available to their community;
(iv) identifying groups that may have low health literacy and greater exposure to cancer risk factors owing to low awareness of ECAC recommendations and poor access to cancer prevention and education.

In meeting these objectives, we were able to support local communities by reporting study findings to primary healthcare (PHC) facilities and local authorities and by providing them with ECAC promotional materials.

## Materials and Methods

### Study Design and Participants

Our project was conducted in a population of volunteers in a commune located in Koło district, Poland, from July to December 2018. It involved surveys and workshops delivered in local rural community centers. The project had been promoted among the PHC staff, on leaflets and posters, through websites, and in social media. While developing and implementing the project, we attempted to use an approach that was novel in two respects. First, it incorporated CBPR to engage multiple community stakeholders. Second, it applied the ECAC in order to identify and fill gaps in health education and promotion.

Our research instrument was an original questionnaire developed in the Poznan University of Medical Sciences. It included sociodemographic questions (gender, age, place of residence, education, occupation, professional status, self-reported economic status) and 24 questions regarding, among other things, health knowledge and its sources, perception of cancer risk factors, health behaviours and lifestyle, as well as the assessment of locally available health education and health promotion activities.

The anonymous questionnaire was delivered at the beginning of each of the seven workshops scheduled every 2 weeks in rural community centers, each time in a different location. Convenience sampling was used; the workshops were open for all willing to participate. The only inclusion criteria were place of residence, age over 18 years, and consent to participate in the study. Completing the questionnaire was voluntary. At the beginning of each meeting, the health educator provided the participants with all project details. She emphasised that the participants had the right to refuse to participate or withdraw from the study at any time, without having to give a reason and without any consequences. During the meetings, participants were trained to calculate their body mass index (BMI) and to perform breast self-exams. They were also given custom pens, cups and cotton bags promoting cancer prevention. Knowledge quizzes were carried out before and after meetings. The participants were invited to comment on the quality of the workshops during a free discussion following each meeting.

### Statistical Analysis

Statistical analyses were conducted using PQStat v 1.6.8 software. A *p*-value below 0.05 was considered significant. Various statistical tests were performed depending on the scale of measure applied, model of analysis used, and the kind of associations searched for. The tests used in the analysis are listed below the tables with the results.

## Results

### The Survey Participants

We collected complete questionnaires from 122 persons (2.3% of the adult inhabitants of the commune) aged from 18 to 78 years (mean age 49.1 ± 15.9), most of them farmers (53.3%). For comparison, 44% of the commune's working inhabitants are employed in farming. Among the respondents, 72.1% (*n* = 88) were women aged 49.3 ± 16.6 years and 27.9% (*n* = 34) were men aged 48 ± 14.5 years. They were mostly inhabitants of small villages (86.1%), while only 13.9% lived in towns with a population under 5,000. The most numerous groups were persons aged 45–64 (41%) and 25–44 years (32%). Most participants had either secondary (35.3%) or vocational education (26.2%). At the same time in the whole adult population of the commune 50.5% were women, 67.6% lived in villages, people aged 45–64 and 25–44 accounted for 31.4 and 36.4%, respectively, 27.7% had secondary education and 26.1% vocational education. The reported economic status was mostly good (58.2%) or average (32.8%). FHC was reported by 54.1% of the participants, it was absent in 36.9%, and 9% were not sure. The reported cancer screening attendance and participation in selected diagnostic tests were described elsewhere (31). Importantly, 82% of the study group indicated the need to participate in our workshops for educational purposes, while the rest mentioned general curiosity as their main reason to participate.

### Health Knowledge and Perception of Health Risk Factors

The participants self-assessed their knowledge on how to take care of their health as good (46.7%) or satisfactory (36.1%) (Table 1). There was a weak positive correlation between the self-assessment results and participants' education (*r* = 0.17; *p* = 0.0042), economic status (r = 0.20; *p* = 0.0391) and occupation (*p* = 0.0374). Self-assessment of knowledge was not associated with FHC (Table 1).

**Table 1:** Self-assessment of health knowledge (*n* = 122).

**Variable**		***n***	**Self-assessment of health knowledge (%)**				
			**Unsatisfactory**	**Satisfactory**	**Good**	**Very good**	***p***
Total		122	3.3	36.1	46.7	4.9	
Age	<25	9	0	37.5	62.5	0	
	25–44	39	2.7	32.4	54.1	10.8	0.0899^a^
	45–64	50	4.5	40.9	50	4.5	*r* = −0.16
	65 or older	24	4.5	50	45.5	0	
Gender	F	88	2.5	36.3	55	6.3	0.0936^b^
	M	34	6.5	48.4	41.9	3.2	
Education	Primary	21	11.8	47.1	35.3	5.9	0.0042^a^
	Lower secondary	2	0	0	100	0	*r* = 0.17
	Vocational	32	3.4	48.3	44.8	3.4	
	Secondary	43	2.4	46.3	51.2	0	
	Higher	24	0	13.6	68.2	18.2	
Professional status	Employed	67	3.3	36.1	52.5	8.2	0.1156^c^
	Retired	46	4.9	41.5	51.2	2.4	
	Unemployed	4	0	100	0	0	
	Students	5	0	20	80	0	
Occupation	Farmers	65	1.8	43.9	50.9	3.5	0.0374^c^
	Other manual workers	20	5.3	52.6	42.1	0	
	Office workers or other specialists	13	16.7	16.7	66.7	0	
	Health professionals	8	0	14.3	71.4	14.3	
	Teachers / educators	7	0	14.3	42.9	42.9	
	Unemployed	9	0	55.6	44.4	0	
Reported economic status	Bad	5	0	60	20	20	
	Average	40	9.4	46.9	37.5	6.3	0.0391^a^
	Good	71	1.5	36.8	58.8	2.9	*r* = 0.20
	Very good	6	0	16.7	66.7	16.7	
Attitude to screening	Does not attend	60	7.3	34.5	50.9	7.3	0.9907^b^
	Attends	53	0	45.8	50	4.2	
Family history of cancer	No	45	4.9	31.7	56.1	7.3	0.4082^b^
	Yes	66	3.3	41.7	51.7	3.3	

*n, group size*;

a *Spearman's rank correlation coefficient*;

b *Chi-squared test for trend*;

>c *Kruskal-Wallis ANOVA*.

Next, we asked what were the most possible reasons perceived as responsible for the development of cancer. The risk factors mentioned the most frequently were genetics (72.1%), stimulants such as alcohol or tobacco (51.5%), and environmental pollution (45.1%) (Table 2). Genetics was the top factor in all groups, although it was mentioned more often by younger (*p* = 0.0001) and better-educated participants (*p* = 0.005). The awareness of the impact of an unhealthy diet was associated with occupation – farmers mentioned this factor significantly less often (*p* = 0.0152). The role of stimulants (alcohol, tobacco) was less frequently recognised by retired compared to employed individuals (*p* = 0.0379). Chronic infections (*p* = 0.0133) were indicated significantly more often by female, better educated and wealthier participants (*p* = 0.0061). The awareness of UV radiation as a risk factor declined with increasing age (*p* = 0.0006). That factor was mentioned more often by employed (*p* = 0.0015) and better educated (*p* = 0.0006) participants as well as by those whose self-reported economic status was better (*p* = 0.0264).

**Table 2:** The most important cancer risk factors according to the participants (*n* = 122).

**Variable**		**Cancer risk factors (%)**												
		***n***	**Genetic factors**	***p***	**Unhealthy diet**	***p***	**Stimulants (alcohol, tobacco)**	***p***	**Chronic infections**	***p***	**UV radiation**	***p***	**Environmental pollution**	***p***
Total		122	72.1		41.8		51.6		26.2		35.2		45.1	
Age	<25	9	88.9	0.0001^b^	55.6	0.185^b^	55.6	0.0933^b^	33.3	0.1286^b^	66.7	<0.0001^b^	44.4	0.1315^b^
	25–44	39	92.3		46.2		66.7		30.8		51.3		59	
	45–64	50	66		40		42		28		34		38	
	65 or older	24	45.8		33.3		45.8		12.5		0		37.5	
Gender	F	88	73.9	0.4923^d^	45.5	0.1884^d^	46.6	0.0726^d^	31.8	0.024^d^	36.4	0.6776^d^	44.3	0.785 ^d^
	M	34	67.6		32.4		64.7		11.8		32.4		47.1	
Education	Primary	21	61.9	0.005^b^	28.6	0.0817^b^	47.6	0.3492^b^	9.5	0.0133^b^	14.3	0.0006^b^	42.9	0.2751^b^
	Lower secondary	2	50		100		50		50		100		100	
	Vocational	32	56.3		34.4		53.1		15.6		25		43.8	
	Secondary	43	76.7		41.9		41.9		34.9		27.9		25.6	
	Higher	24	95.8		58.3		70.8		37.5		75		79.2	
Professional status	Employed	67	80.6	0.0686^e^	44.8	0.5478^e^	62.7	0.0379^e^	23.9	0.2266^e^	44.8	0.0015^e^	55.2	0.0565^e^
	Retired	46	60.9		34.8		37		28.3		19.6		30.4	
	Unemployed	4	50		50		50		0		0		50	
	Students	5	80		60		40		60		80		40	
Occupation	Farmers	65	66.2	0.6333^e^	30.8	0.0152^e^	47.7	0.9109^e^	27.7	0.1478^e^	33.8	0.3828^e^	44.6	0.0847^e^
	Other manual workers	20	85		35		55		10		25		20	
	Office workers or other specialists	13	76.9		76.9		61.5		15.4		30.8		61.5	
	Health professionals	8	75		50		62.5		37.5		37.5		62.5	
	Teachers / educators	7	85.7		71.4		57.1		57.1		71.4		71.4	
	Unemployed	9	66.7		55.6		44.4		33.3		44.4		44.4	
Economic status	Bad	5	80	0.239^b^	0	0.0004^b^	80	0.6266^b^	0	0.0061^b^	20	0.0264^b^	20	0.6023^b^
	Average	40	60		25		40		15		25		47.5	
	Good	71	78.9		52.1		56.3		32.4		39.4		45.1	
	Very good	6	66.7		66.7		50		50		66.7		50	
Attitude to screening	Does not attend	60	78.3	0.2111^d^	40	0.8705^d^	56.7	0.1591^d^	16.7	0.0553^d^	36.7	0.6084^d^	46.7	0.7274^d^
	Attends	53	67.9		41.5		43.4		32.1		32.1		43.4	
Family history of cancer	No	45	61.9	0.1022^d^	45.2	0.9085^d^	54.8	0.8524^d^	21.4	0.216^d^	35.7	0.7905^d^	38.1	0.2847^d^
	Yes	66	76.5		44.1		52.9		32.4		38.2		48.5	

*n, group size*;

b *Chi-squared test for trend*;

d *Pearson's chi-squared test*;

e *Fisher's exact test*.

Only 42% of the respondents were familiar with the healthy eating pyramid published by the National Food and Nutrition Institute of Poland (36). Furthermore, only 8.2% of the participants (all female) actually knew all the principles of healthy eating. The knowledge declined with decreasing education (*p* = 0.0333) and was affected by occupation, with manual workers and farmers being the least knowledgeable (Table 3). FHC was not associated with these results (Table 3). The best-known principles were eating at regular intervals (78.7%), and consuming more vegetables than fruit (53.3%). Only 23.8% realised the importance of limiting the consumption of red meat.

**Table 3:** The knowledge of the healthy eating pyramid (*n* = 122).

**Variable**		***n***	**Reported knowledge of the pyramid (%)**				**Correct indication of the rules of healthy eating (%)**
			**No**	**Yes**	***p***	**I don't know**	
Total		122	34	42		24	8.2
Age	<25	9	22.2	55.6	0.072^b^	22.2	11.1
	25–44	39	17.9	53.8		28.2	12.8
	45–64	50	52	30		18	4
	65 or older	24	29.2	41.7		29.2	8.3
Gender	F	88	28.4	48.9	0.0269^d^	22.7	11.4
	M	34	50	23.5		26.5	0
Education	Primary	21	42.9	28.6	0.0333^b^	28.6	0
	Lower secondary	2	0	100		0	0
	Vocational	32	43.8	34.4		21.9	6.3
	Secondary	43	37.2	37.2		25.6	4.7
	Higher	24	12.5	66.7		20.8	25
Professional status	Employed	67	32.8	41.8	0.7664^e^	25.4	9
	Retired	46	39.1	37		23.9	6.5
	Unemployed	4	25	50		25	0
	Students	5	20	80		0	4
Occupation	Farmers	65	41.5	36.9	0.0051^e^	21.5	4.6
	Other manual workers	20	45	15		40	0
	Office workers or other specialists	13	23.1	38.5		38.5	7.7
	Health professionals	8	12.5	75		12.5	25
	Teachers / educators	7	0	100		0	42.9
	Unemployed	9	22.2	66.7		11.1	11.1
Reported economic status	Bad	5	40	20	0.0742^b^	40	0
	Average	40	47.5	32.5		20	7.5
	Good	71	25.4	47.9		26.8	33.3
	Very good	6	50	50		0	0
Attitude to screening	Does not attend	60	33.3	36.7	0.1889^d^	30	5
	Attends	53	32.1	50.9		17	7.5
Family history of cancer	No	45	42.9	38.1	0.1913^d^	19	4.4
	Yes	66	26.5	45.6		27.9	12.1

*n, group size*;

b *Chi-squared test for trend*;

d *Pearson's chi-squared test*;

e *Fisher's exact test*.

The main sources of knowledge on how to take care of one's health were the media (78%), health professionals and educational materials available at outpatient clinics (63%), as well as other sources – family, friends, schools or universities (37%). The details are presented in Table 4.

**Table 4:** The sources of health knowledge (*n* = 122).

**Variable**		**n**	**The main source of knowledge (%)**					
			**TV, Internet, press, radio**	***p***	**Professionals and materials at clinics**	***p***	**Other sources (school, family, friends)**	***p***
Total		122	78		63		37	
Age	<25	9	100	0.0638^b^	44.4	0.0874^b^	77.8	0.0319^b^
	25–44	39	79.5		48.7		43.6	
	45–64	50	78		80		24	
	65 or older	24	66.7		58.3		37.5	
Gender	F	88	85.2	0.0016^d^	60.2	0.2876^d^	34.1	0.3034^d^
	M	34	58.8		70.6		44.1	
Education	Primary	21	61.9	0.0159^b^	71.4	0.5016^b^	42.9	0.5321^b^
	Lower secondary	2	100		50		100	
	Vocational	32	65.6		65.6		34.4	
	Secondary	43	90.7		55.8		30.2	
	Higher	24	83.3		66.7		41.7	
Professional status	Employed	67	80.6	0.2573^e^	64.2	0.7393^e^	38.8	0.164^e^
	Retired	46	73.9		63		30.4	
	Unemployed	4	50		75		25	
	Students	5	100		40		80	
Occupation	Farmers	65	75.4	0.3528^e^	69.2	0.1621^e^	33.8	0.7321^e^
	Other manual workers	20	65		45		45	
	Office workers or other specialists	13	92.3		46.2		38.5	
	Health professionals	8	87.5		87.5		25	
	Teachers / educators	7	100		71.4		28.6	
	Unemployed	9	77.8		55.6		55.6	
Economic status	Bad	5	20	0.0734^b^	80	0.1258^b^	20	0.8216^b^
	Average	40	80		70		42.5	
	Good	71	80.3		59.2		35.2	
	Very good	6	83.3		50		33.3	
Attitude to screening	Does not attend	60	76.7	0.8818^d^	51.7	0.0046^d^	45	0.0231^d^
	Attends	53	75.5		77.4		24.5	
Family history of cancer	No	45	73.8	0.3829^d^	59.5	0.481^d^	38.1	0.8885^d^
	Yes	66	80.9		66.2		36.8	

*n, group size*;

b *Chi-squared test for trend*;

d *Pearson's chi-squared test*;

e *Fisher's exact test*.

### Health Behaviours and Lifestyle

When asked if they took care of their health, the participants usually answered ‘rather yes' (71.3%). The affirmative answers were associated with economic status (*r* = 0.25; *p* = 0.0065) and screening test attendance (*p* = 0.0047). The test attendees also more often claimed to follow the WHO's recommendations regarding physical activity (PA) (*p* = 0.052) and smoked less often (*p* = 0.0029). Generally, 63.1% said they followed the PA recommendations, but 35.3% were not able to determine their weekly PA. As many as 41.2% of men and 9.1% of women were current smokers. The proportion was much higher in men (*p* < 0.0001) and decreased with better education (*p* = 0.0089). It was also associated with professional status (*p* = 0.008), occupation (*p* = 0.0163) and economic status (p = 0.003). Abstaining from alcohol was reported by 23.8% of the participants. FHC did not differentiate the responses in the area of health behaviours (Table 5).

**Table 5:** The reported taking care of one's health and selected health behaviours (*n* = 122).

			**Reported taking care of one's health (%)**										
**Variable**		***n***	**Definitely not**	**Rather not**	**Rather yes**	**Definitely yes**	***p***	**Followers of WHO recommendations on PA (%)**	***p***	**Smokers** **(%)**	***p***	**Teetotallers** **(%)**	***p***
Total		122	2.5	13.1	71.3	5.7		63.1		18		23.8	
Age	<25	9	0	0	100	0		88.9	0.9868^b^	33.3	0.1664^b^	22.2	0.3413^b^
	25–44	39	5.1	17.9	69.2	5.1	0.514^a^	61.5		12.8		25.6	
	45–64	50	2	12	72	6	r = 0.06	60		28		14	
	65 or older	24	0	12.5	62.5	8.3		62.5		0		41.7	
Gender	F	88	2.3	10.2	71.6	6.8	0.1659^b^	62.5	1^e^	9.1	<0.0001^d^	26.1	0.3233^d^
	M	34	2.9	20.6	70.6	2.9		64.7		41.2		17.6	
Education	Primary	21	4.8	33.3	47.6	4.8		52.4	0.8188^b^	33.3	0.0089^b^	28.6	0.6044^b^
	Lower secondary	2	0	0	100	0	0.2516^a^	100		0		50	
	Vocational	32	3.1	9.4	68.8	9.4	*r* = 0.11	56.3		25		18.8	
	Secondary	43	0	4.7	81.4	4.7		67.4		14		25.6	
	Higher	24	4.2	16.7	75	4.2		70.8		4.2		20.8	
Professional status	Employed	67	1.5	14.9	74.6	3	0.1653^c^	64.2	1^e^	22.4	0.008^e^	14.9	0.0366^e^
	Retired	46	2.2	10.9	65.2	10.9		63		8.7		32.6	
	Unemployed	4	25	25	50	0		25		75		50	
	Students	5	0	0	100	0		80		0		40	
Occupation	Farmers	65	1.5	10.8	72.3	6.2	0.6864^c^	55.4	0.0514^e^	16.9	0.0163^e^	23.1	0.4348^e^
	Other manual workers	20	0	20	70	5		85		40		25	
	Office workers or other specialists	13	0	7.7	76.9	0		84.6		0		23.1	
	Health professionals	8	0	12.5	75	12.5		62.5		0		0	
	Teachers / educators	7	14.3	28.6	42.9	14.3		42.9		0		28.6	
	Unemployed	9	11.1	11.1	77.8	0		55.6		33.3		44.4	
Economic status	Bad	5	20	40	20	20		60	0.497^b^	60	0.003^b^	60	0.24^b^
	Average	40	5	17.5	57.5	2.5	0.0065^a^	45		27.5		25	
	Good	71	0	8.5	84.5	5.6	*r* = 0.25	71.8		9.9		19.7	
	Very good	6	0	16.7	50	16.7		83.3		16.7		33.3	
Attitude to screening	Does not attend	60	5	16.7	73.3	1.7	0.0047^b^	55	0.052^e^	26.7	0.0029^d^	26.7	0.6209^d^
	Attends	53	0	7.5	71.7	11.3		69.8		5.7		22.6	
Family history of cancer	No	45	0	16.7	64.3	9.5	0.882^b^	61.9	1^e^	19	0.7966^d^	23.8	0.8244^d^
	Yes	66	1.5	10.3	77.9	4.4		69.1		16.2		26.5	

*n, group size*;

a *Spearman's rank correlation coefficient*;

b *Chi-squared test for trend*;

c *Kruskal-Wallis ANOVA*;

d *Pearson's chi-squared test*;

e *Fisher's exact test*.

When asked to describe their lifestyles (multiple answers allowed), participants usually called it stressful (47.5%), fast-paced/intense (44.3%), and chaotic (23%). Only 6.6% described it as healthy. Lifestyle differed depending on age and professional status (Table 6). When asked about barriers preventing them from leading a healthy lifestyle, the participants usually said there were no such barriers (36.9%), or mentioned lack of time (30.3%) and bad habits (27.9%). FHC did not differentiate the participants' responses regarding lifestyle and barriers (Table 6).

**Table 6:** Lifestyle (*n* = 122).

			**Lifestyle (%)**				**Main barriers to healthy lifestyle (%)**					
**Variable**		**n**	**Peaceful harmonious well-organised healthy**	***p***	**Fast-paced intense chaotic stressful unhealthy**	***p***	**None**	***p***	**Lack of** **time**	***p***	**Bad** **habits**	***p***
Total		122	35.2		73.8		36.9		30.3		27.9	
Age	<25	9	33.3	0.0053^b^	77.8	0.0381^b^	11.1	0.0452^b^	66.7	<0.0001^b^	66.7	0.0012^b^
	25–44	39	15.4		89.7		28.2		56.4		35.9	
	45–64	50	42		64		46		14		22	
	65 or older	24	54.2		66.7		41.7		8.3		12.5	
Gender	F	88	36.4	0.833^e^	75	0.65^e^	37.5	0.8209^d^	29.5	0.7623^d^	28.4	0.8305^d^
	M	34	32.4		70.6		35.3		32.4		26.5	
Education	Primary	21	38.1	0.1242^b^	76.2	0.3173^b^	38.1	0.6307^b^	4.8	0.0001^b^	14.3	0.0909^b^
	Lower secondary	2	50		50		0		50		50	
	Vocational	32	46.9		62.5		43.8		18.8		28.1	
	Secondary	43	34.9		74.4		37.2		32.6		25.6	
	Higher	24	16.7		87.5		29.2		62.5		41.7	
Professional status	Employed	67	17.9	<0.0001^e^	85.1	0.0084^e^	34.3	0.0491^e^	44.8	<0.0001^e^	34.3	0.0392^e^
	Retired	46	58.7		58.7		47.8		6.5		17.4	
	Unemployed	4	25		75		0		25		0	
	Students	5	60		60		0		60		60	
Occupation	Farmers	65	33.8	0.9138^e^	75.4	0.9555^e^	38.5	0.2294^e^	27.7	0.2357^e^	21.5	0.4565^e^
	Other manual workers	20	35		70		40		20		40	
	Office workers or other specialists	13	46.2		69.2		46.2		23.1		23.1	
	Health professionals	8	25		75		37.5		62.5		37.5	
	Teachers / educators	7	28.6		85.7		42.9		42.9		42.9	
	Unemployed	9	44.4		66.7		0		44.4		33.3	
Economic status	Bad	5	40	0.3019^b^	60	0.2667^b^	20	0.0343^b^	0	0.024^b^	60	0.1356^b^
	Average	40	42.5		70		25		20		27.5	
	Good	71	31		76.1		43.7		38		28.2	
	Very good	6	33.3		83.3		50		33.3		0	
Attitude to screening	Does not attend	60	28.3	0.2878^d^	78.3	0.5547^b^	30	0.0382^d^	36.7	0.0634^d^	26.7	0.7951^d^
	Attends	53	37.7		73.6		49.1		20.8		24.5	
Family history of cancer	No	45	40.5	0.3869^d^	71.4	0.5553^d^	42.9	0.4277^d^	28.6	0.6767^d^	19	0.0942^d^
	Yes	66	32.4		76.5		35.3		32.4		33.8	

*n, group size*;

b *Chi-squared test for trend*;

d *Pearson's chi-squared test*;

e *Fisher's exact test*.

### The Assessment of Health Education and Health Promotion Activities

Locally available health education and promotion activities were often rated as unsatisfactory (41%; see Table 7). As many as 64.8% of the respondents had not been informed about cancer prevention in their PHC clinics. The proportions were particularly high in men (79.4%) (*p* = 0.0329), other manual workers (80%) and farmers (73.8%). Notably, it was farmers who were the least satisfied with the activities (Table 7).

**Table 7:** The assessment of health education and health promotion activities (*n* = 122).

**Variable**		***n***	**Assessment of health education and health promotion (%)**					**Have you ever been informed about cancer prevention at a primary care clinic in the commune? (%)**		
			**Unsatisfactory**	**Satisfactory**	**Good**	**Very good**	***p***	**No**	**Yes**	***p***
Total		122	41	21.3	19.7	3.3		64.8	20.5	
Age	<25	9	33.3	11.1	22.2	11.1	0.882^a^	44.4	33.3	0.6297^b^
	25–44	39	43.6	25.6	15.4	0		74.4	17.9	
	45–64	50	42	20	26	4	*r* = 0.015	60	22	
	65 or older	24	37.5	20.8	12.5	4.2		66.7	16.7	
Gender	F	88	43.2	15.9	22.7	3.4	0.8145^b^	59.1	25	0.0329^d^
	M	34	35.3	35.3	11.8	2.9		79.4	8.8	
Education	Primary	21	23.8	19	28.6	9.5		57.1	14.3	0.232^b^
	Lower secondary	2	50	0	50	0	0.202^a^	0	50	
	Vocational	32	40.6	31.3	6.3	3.1	*r* = −0.13	75	9.4	
	Secondary	43	48.8	11.6	25.6	2.3		67.4	23.3	
	Higher	24	41.7	29.2	16.7	0		58.3	33.3	
Professional status	Employed	67	35.8	29.9	17.9	1.5	0.6357^c^	65.7	19.4	0.3574^e^
	Retired	46	47.8	10.9	19.6	4.3		31 67.4	21.7	
	Unemployed	4	50	125	25	0		75	0	
	Students	5	40	0	40	20		20	40	
Occupation	Farmers	65	50.8	20	13.8	1.5	0.1056^c^	73.8	10.8	<0.0001^e^
	Other manual workers	20	30	25	5	10		80	5	
	Office workers or other specialists	13	38.5	15.4	38.5	0		53.8	38.5	
	Health professionals	8	12.5	25	50	0		12.5	87.5	
	Teachers / educators	7	14.3	42.9	28.6	0		42.9	42.9	
	Unemployed	9	44.4	11.1	33.3	11.1		44.4	22.2	
Economic status	Bad	5	0	40	20	0		40	40	0.7379^b^
	Average	40	37.5	20	20	5	0.2588^a^	65	12.5	
	Good	71	45.1	21.1	19.7	2.8	*r* = −0.11	66.2	22.5	
	Very good	6	50	16.7	16.7	0		66.7	33.3	
Attitude to screening	Does not attend	60	41.7	25	13.3	3.3	0.2069^b^	73.3	11.7	0.0043^d^
	Attends	53	37.7	17	28.3	3.8		52.8	34	
Family history of cancer	No	45	40.5	26.2	21.4	0	0.7456^b^	61.9	21.4	0.8645^d^
	Yes	66	41.2	20.6	19.1	4.4		64.7	20.6	

*n, group size*;

a *Spearman's rank correlation coefficient*;

b *Chi-squared test for trend*;

c *Kruskal-Wallis*;

d *Pearson's chi-squared test*;

e *Fisher's exact test*.

## Discussion

ECAC recommendations should be applied in health education throughout Europe. A recent European survey study on ECAC awareness showed that 21% of Poles heard of the ECAC. In fact, 80% of the Polish respondents admitted they “learned anything new about cancer prevention” after reading the ECAC recommendations. It was the biggest proportion among all the countries surveyed (6), which seems to suggest there are big gaps in Poles' cancer knowledge. Our results demonstrated not only gaps in the study group's knowledge, but also the respondents' low ratings of education on cancer prevention provided at PHC clinics. Finally, our observations indicate widespread dissatisfaction with accessibility of health education and promotion in the community as well as an urgent need for improvement in this area. It is even more alarming when we realise that although the participants were aware of the importance of genetic risk factors for cancer, FHC did not change their knowledge or health-related behaviours.

### Health Knowledge and Awareness of Cancer Risk Factors

It is important to explore the beliefs and knowledge of the target population in order to ensure the effectiveness of cancer prevention initiatives (37). There are studies demonstrating that the knowledge of cancer risk factors is limited (7, 38–40). Moreover, 44.4% of men and 37.7% of women in Poland had never come across the notion of a risk factor (41). Awareness of particular risk factors can vary widely, depending on the disease. One study showed the highest awareness of risk factors for lung cancer and melanoma, and the lowest – for bowel, breast, cervical and prostate cancers (38). Participants of another study were most familiar with such risk factors as pollution, smog, artificial food additives, smoking, stress, and bad diet (42).

According to our respondents, the biggest risk factor for cancer is genetics. In a recent Turkish study, 76.4% of the respondents expressed a belief that cancer is hereditary (43). The beliefs of our respondents do not fully reflect the relative importance of cancer risk factors in Europe. It is estimated that hereditary cancers constitute a small proportion of new cancer cases and that approximately 5% of all cancers are strictly hereditary (8). In European countries, the most important risk factors are smoking, unhealthy diet, alcohol consumption, overweight and obesity, as well as infections, occupational exposures, and environmental pollution (3, 8, 11). The risk factors that are more prevalent in rural areas include excessive sun exposure, higher smoking rates, low smear test attendance (23, 30, 44), excessive weight (41, 45), and chemical carcinogens (25).

UV radiation was not commonly recognised as a risk factor by our respondents despite high occupational exposure in this population and repeated suggestions for the need to educate high risk groups (23, 46). While the risk actually increases with age, we found older people less aware of it. Considering the insufficient skin cancer prevention behaviours in rural areas (47, 48), the message about the need for UV protection should be reinforced.

Next, our study participants underestimated the impact of unhealthy diet. Poor nutrition is strongly associated with lifestyle diseases, including cancer (2, 3, 7). To choose a healthy diet, people need to at least know the current dietary recommendations, which are regularly published in most countries (49). Nutrition education in industrialised countries is common (14), but clear messages about healthy eating are not communicated to the general public and knowledge about a healthy diet is unsatisfactory (49). In our study, a big proportion of the respondents admitted they did not know the food pyramid and its principles. These observations are partially corroborated by the Kantar report of 2017 (42) and are generally consistent with other findings showing that nutrition knowledge depends on gender, education level, and socioeconomic status (49). Nutrition knowledge is an integral part of health literacy (49, 50). Although such knowledge alone is not sufficient to protect health (14, 15), a positive correlation between nutrition knowledge and dietary intake has been observed (14, 51, 52). Awareness of specific gaps in the knowledge of dietary recommendations may allow for better planning of educational initiatives (15).

In our study group, chronic infections were rarely indicated as a risk factor. While infections are strong risk factors for certain cancers and up to 16% of new cancer cases worldwide can be attributed to them, they seem to be of decreasing importance in highly developed countries (1, 3). One of the reasons is the availability of human papillomavirus (HPV) vaccines (2). In Poland as of October 2021, this vaccination was not available under the public health insurance (53). Therefore, there are no conditions to effectively implement the ECAC recommendations for its use.

In our study, health knowledge did not increase with age or with listing ‘professionals and materials at clinics' as sources of health knowledge. This is consistent with the finding that ECAC awareness decreases with age (6), which should be addressed given the epidemiology of most cancers (1, 54).

Given the above knowledge gaps, we noticed a pressing need to promote the ECAC recommendations. One of the factors hindering the promotion of the ECAC is the one-size-fits-all approach to the general population (6). Our solution provided an opportunity to adjust the strength of particular ECAC recommendations so as to promote the right messages at the right time and to the right audience. It follows from pre- and post-workshop knowledge quizzes that the workshops increased the respondents' knowledge by 33.33% on average.

### Health Behaviours and Lifestyle

According to current data, only around half of general Polish population recognizes their own behaviours as the most important factor influencing health (41). Unhealthy behaviours are observed more often among farmers than in the urban population (19). Most of our respondents, when asked if they were taking care of their health, answered “rather yes” (71.3%) or “definitely yes” (5.7%). In contrast, 50% of participants of the 2017 Kantar study answered “rather yes”, while as many as 28% – “definitely yes” (42). Taking better care of one's health was more often reported by the oldest respondents (42), which we did not observe.

As regards PA, its relationship with cancer risk reduction is complex and its beneficial effect results mainly from the prevention of overweight and obesity (1). When our workshops were taking place, adults were advised to have at least 150 min of moderate activity or 75 min of vigorous activity per week (16, 55). 63.1% of our participants claimed to meet these recommendations. The PA of our respondents was therefore slightly higher than in other studies (56, 57), some of which suggest that PA levels in rural areas are unsatisfactory (57). Unfortunately, 35.3% of our respondents were not able to state their weekly PA levels. It follows that the assessment of PA levels in rural settings may be difficult.

Smoking is the leading cause of preventable cancer cases (58, 59). As for alcohol, the ECAC recommends teetotalism or at least limiting consumption (2, 16). Among our participants, ‘stimulants (alcohol, tobacco)' was indicated as the second most important risk factor for cancer. The proportion of alcohol abstainers (23.8%) was slightly higher than previously observed in the general population (42). This may be due to the fact that our sample was feminised; it had been previously reported that women tended to drink less alcohol than men (59). As for smoking, we corroborated the available research showing that it was correlated with education and gender, although we recorded a higher percentage of male smokers and a lower percentage in females than previously reported (42, 59).

### Family History of Cancer vs. Health Knowledge and Cancer Prevention

We found no significant differences in lifestyle, health behaviours, taking care of one's health, knowledge and its sources, or perception of cancer risk factors between the groups with and without FHC. FHC may reflect genetic risks as well as behavioural and environmental risks shared by family members (60, 61). There are arguments for using FHC as a significant motivational tool in cancer prevention (60, 61). Some cancers can be prevented even among people with FHC through behavioural modifications and participation in screening (60, 61). Some reports indicate that persons with FHC are more likely to participate in screening, but otherwise do not have better health behaviours than those without FHC (62, 63). The reasons are diverse (60). We need more research on the impact of FHC on cancer prevention knowledge and behaviours. Feedback from our respondents suggests that, unfortunately, people with FHC are not given more recommendations about cancer prevention by PHC facilities.

### The Assessment of Health Education and Health Promotion Activities

Preventive counselling is often neglected by health care providers (32, 64, 65). It has been shown that more than 60% of Polish general practitioners (GPs) do not assess patients' lifestyles during consultations, although 64% believe they are obliged to provide health education; only 30% of GPs think that their knowledge in this area is sufficient (32). It translates to knowledge gaps among patients: for example most PHC patients in central Poland did not know about colorectal cancer prevention, and their knowledge about diet and physical activity was insufficient (65).

Health education and promotion in PHC is particularly needed in rural areas, because the law on farmers' occupational health care in Poland makes it difficult for this professional group to have periodic health checks (21). Among our participants, the group that was particularly critical of the available health education and promotion activities was farmers, who were also considerably less often informed about cancer prevention at their local clinics. In addition, it was men who were less frequently informed about cancer prevention in PHC, although their knowledge and behaviours regarding cancer risk factors are found to be worse than women's (65), and their willingness to reduce cancer risk through lifestyle change is also lower (6). Importantly, the incidence and mortality of preventable cancers are higher in men than in women (1, 4). Our results also contradict previous findings that cancer prevention counselling intensifies with the growing age of patients (65).

As many as 64.8% of our respondents indicated that they had never received information about cancer prevention at their PHC clinic. On the whole, the assessment of the available health education and promotion activities was poor. The workshops thus became an opportunity to fill some gaps (e.g., we explained breast cancer prevention, taught breast self-exams and BMI calculation, and offered assistance in making appointments for cancer prevention programmes). We promoted the ECAC among personnel at PHC clinics and pharmacies through meetings and the provision of educational materials.

### Limitations and Strengths

Our study has a few limitations. Firstly, multiple locations and extensive advertising of the workshops were intended to give everyone an equal chance to participate, but the sample cannot be considered representative. Due to a limited budget, we were not able to reach a large sample. Also, women were overrepresented, and gender is known to influence both health knowledge and health behaviours.

Another limitation was that we did not verify information about FHC or reported participation in cancer prevention with patients' medical records or cancer registries. The collected responses may carry a recall bias.

The strength of our project is a new approach to promote the ECAC. We received very positive feedback from our participants about the quality of the workshops. In our opinion, the project promotes a better understanding of the ECAC while simultaneously reducing social inequalities in cancer prevention, which is in line with one of the ECAC guiding principles (12). We were able to adjust the strength of each ECAC recommendation to best fit the target audience.

## Conclusions

Our project was CBPR-based and partly intervention-oriented, which is why it was meant to reach and support a small and very specific population rather than a large representative sample. Still, we believe that its results allowed us to reach conclusions which may be useful for future large-scale interventions. This is what we concluded regarding (i) our participants' health knowledge, (ii) their health behaviours, (iii) their assessment of the currently available health education and promotion opportunities, and (iv) the high-risk groups within the studied community.

(i) As regards the knowledge of our participants, the overrating of genetic determinants of cancer coincided with the alarmingly limited awareness of modifiable lifestyle-related risk factors. Since the ECAC should be promoted with specific needs of each population in mind, the most pressing educational needs in populations similar to ours are: the prevention of UV exposure, nutrition education, combating tobacco addiction, and encouraging participation in preventive screenings.
(ii) When it comes to health behaviours, most of our study group asserted that they were taking care of their health and it was reflected in relatively big proportions of respondents who reported meeting PA recommended levels, being smoke-free, and abstaining from alcohol. However, our sample was feminised and some of the healthy behaviours tended to be more prevalent in women. Neither older age nor FHC led to healthier practices in our study group. Thus, it seems that interventions aiming to promote healthy lifestyle should focus on men, the elderly, and people with FHC.
(iii) Feedback from our respondents suggests that, unfortunately, health education and health promotion opportunities available to them are insufficient. Moreover, the respondents with FHC are not provided with more intense cancer counselling at PHC facilities. Health professionals should consider communicating to all patients, especially to those with FHC, that we can all have a positive impact on our health by following the ECAC. This could increase the chance of breaking the intergenerational transmission of anti-health behaviours.
(iv) Given the findings above, it seems that ECAC-based health education is very urgent especially among farmers, men, people with FHC, and older people. In these groups, the high prevalence of cancer risk factors coincides with being overlooked as health education targets.

We provided local PHC staff and authorities with the conclusions described above. Among PHC professionals, we also promoted the ECAC as a perfect tool for supporting their health education efforts.

The project that we designed and are presenting here integrated the activities of a local community, a non-governmental organization, academics, health professionals and authorities. We strongly recommend such an approach, as it combines multiple perspectives in order to better diagnose and target specific communities. Thus, we can draw the attention of decision-makers to local health promotion needs, which is a good starting point for improving the situation. What is more, we, as university teachers, may also benefit from the project and better educate undergraduate and postgraduate medical professionals on cancer education and prevention. We believe that our project and its results may help guide the future development of educational initiatives in other regions with similar conditions.

## Data Availability Statement

The raw data supporting the conclusions of this article will be made available by the authors, without undue reservation.

## Ethics Statement

Ethical review and approval was not required for the study on human participants in accordance with the local legislation and institutional requirements. Written informed consent for participation was not required for this study in accordance with the national legislation and the institutional requirements.

## Author Contributions

MK and EC: conceptualisation and visualisation. MK: data curation, funding acquisition, investigation, methodology, project administration, and writing - original draft. MK, EC, AL, and BW: formal analysis and resources. BW: statistical analysis. MK and BW: software. EC and AL: supervision. MK, EC, and AL: writing - review and editing. All authors read and approved the final manuscript.

## Funding

This health intervention undertaken as part of this research was funded by the Polish Cancer League (Polska Liga Walki z Rakiem), grant number 5/2018 in the 2018 Onkogrant competition. The funders had no role in the design of the study; in the collection, analyses, or interpretation of data; in the writing of the manuscript, or in the decision to publish the results.

## Conflict of Interest

The authors declare that the research was conducted in the absence of any commercial or financial relationships that could be construed as a potential conflict of interest.

## Publisher's Note

All claims expressed in this article are solely those of the authors and do not necessarily represent those of their affiliated organizations, or those of the publisher, the editors and the reviewers. Any product that may be evaluated in this article, or claim that may be made by its manufacturer, is not guaranteed or endorsed by the publisher.

## Abbreviations

ECAC: European Code Against Cancer
FHC: family history of cancer
EU: European Union
PHC: primary health care
CBPR: community-based participatory research
BMI: body mass index
PA: physical activity
HPV: human papillomavirus
GP: general practitioner.
